# The effectiveness of aseptic non-touch technique audit cycle implementation on reducing the surgical site infection at EMC Group Hospital

**DOI:** 10.1017/ash.2025.140

**Published:** 2025-09-03

**Authors:** Siti Indriani

**Keywords:** Aseptic Non-touch Technique, Audit Cycle, Aseptic Technique, Surgical Site Infection

## Abstract

**Background:** The surgical site infection (SSI) event is still the main cause of morbidity and increases the length of stay (LOS) in a hospital, costs, and deaths. One of the causes of SSI is nurses’ non-compliance with aseptic techniques. Exposure can be increased and variability in aseptic techniques can be minimized by implementing the aseptic non-touch technique (ANTT) strategy. This research investigated the effectiveness of implementing the ANTT method on reducing the number of SSI events in postoperative patients. **Methods:** This research used a pragmatic evaluation method with a mixed methods approach. The ANTT method was implemented through five stages i.e., planning, launching, educating, assessing, and monitoring. Data were collected using pre/post-questionnaires, structured interviews, and audit observations via the electronic audit. This research was conducted for 24 months and involved 138 nurses from three hospitals. **Results:** The mean compliance rate of aseptic techniques after implementing the ANTT method was 93%, with the glove use at 92%, contaminated gloves at 0%, aseptic area at 84%, contaminated aseptic area at 0%, disinfected procedure trays at 75%, protected keypart at 84%, contaminated keyparts by hands/ equipment at 2.6%, and hand hygiene after gloves at 98%. The rate of SSI events dropped from 0.3% to 0.1% in 12 months. There was a significant difference between the pre and post-implementation of the ANTT (P-value < 0.001). Moreover, implementing the ANTT method effectively reduced the SSI events in postoperative patients (P-value < 0.001). **Conclusions:** The implementation of ANTT improved compliance with safe and effective aseptic techniques. The ANTT compliance audit results reflect a reduction in the incidence of SSIs, thereby reducing costs and LOS in postoperative patients. The program will be expanded to five other group hospitals.

